# Supplementary material for: Development of a Mucoadhesive Vehicle Based on Lyophilized Liposomes for Drug Delivery through the Sublingual Mucosa
Source: Pharmaceutics. 2022 Jul 19;14(7):1497. doi: 10.3390/pharmaceutics14071497 (PMC9317145; doi:10.3390/pharmaceutics14071497)
Supplement: Supplementary file 1 [file pharmaceutics-14-01497-s001.zip › pharmaceutics-1809182-supplementary.pdf]

Telstar

Recipe report

Batch: 2111221512_Lote1 **Start:** 22/11/2021 15:13:06 **End:** 25/11/2021 17:02:44

User:: ma **User group::** Maintenance **Computer::** LYOSUITELAB

Recipe Recetaparaprotocolo1 **Version:** 1 **Saved on:** 22/11/2021 15:07:19 **Saved by:** ma

Comment: Receta para Protocolo 1

Step	Process	Temperature (°C)	Vacuum (µbar a)	HH	MM	Temperature limit	Transition by product temperature	Vacuum limit	Safety cooling	Pressure rise test	Vacuum control
1	Shelves temperature	10.0									
2	Freezing	10.0		0	30						
3	Freezing	-60.0		1	0	✓					
4	Freezing	-60.0		3	0						
5	Freezing	-30.0		0	20	✓					
6	Condenser preparation			0	10						
7	Chamber vacuum		100								
8	Primary drying	-30.0	100	0	20						
9	Primary drying	-30.0	100	24	0					✓	
10	Secondary drying	10.0	0	0	20	✓					
11	Secondary drying	10.0	0	12	0					✓	
12	Secondary drying	18.0	0	0	5	✓					
13	Secondary drying	18.0	0	6	0					✓	
14	End of cycle										
15											
16											
17											
18											

Figure S1. Recipe for cycle 1 used for the ramp temperature approach.

Telstar

Recipe report

Batch: 2201131225_13enero2022 **Start:** 13/01/2022 12:25:18 **End:** 18/01/2022 11:37:31

User:: ma **User group::** Maintenance **Computer::** LYOSUITELAB

Recipe riskedcycle **Version:** 1 **Saved on:** 13/01/2022 12:22:07 **Saved by:** ma

Comment: Receta para Protocolo 1

Step	Process	Temperature (°C)	Vacuum (µbar a)	HH	MM	Temperature limit	Transition by product temperature	Vacuum limit	Safety cooling	Pressure rise test	Vacuum control
1	Shelves temperature	5.0									
2	Freezing	5.0		0	30						
3	Freezing	-45.0		1	0	✓					
4	Freezing	-45.0		3	0						
5	Freezing	-25.0		0	20	✓					
6	Condenser preparation			0	10						
7	Chamber vacuum		100								
8	Primary drying	-25.0	100	0	20	✓					
9	Primary drying	-25.0	100	4	0						
10	Primary drying	-22.0	25	0	10	✓					
11	Primary drying	-22.0	25	4	0						
12	Primary drying	-20.0	25	0	10	✓					
13	Primary drying	-20.0	25	4	0						
14	Primary drying	-17.0	25	0	10	✓					
15	Primary drying	-17.0	25	4	0						
16	Primary drying	-15.0	25	0	10	✓					
17	Primary drying	-17.0	25	4	0					✓	
18	Secondary drying	15.0	0	0	30	✓					

Figure S2. Recipe for cycle 2 used for the ramp temperature approach.

Telstar

Recipe report

Batch: 2203081732_lotecompletode **Start:** 08/03/2022 17:32:57 **End:** 10/03/2022 20:37:14

User:: ma **User group::** Maintenance **Computer::** LYOSUITELAB

Recipe mucoadhesivosbucales **Version:** 7 **Saved on:** 08/03/2022 16:01:49 **Saved by:** ma

Comment: receta pra mucoadhesivos bucales

Step	Process	Temperature (°C)	Vacuum (µbar a)	HH	MM	Temperature limit	Transition by product temperature	Vacuum limit	Safety cooling	Pressure rise test	Vacuum control
1	Shelves temperature	5.0									
2	Freezing	5.0		0	30						
3	Freezing	-40.0		1	0	✓					
4	Freezing	-40.0		4	0						
5	Freezing	-20.0		0	20	✓					
6	Condenser preparation			0	10						
7	Chamber vacuum		100								
8	Primary drying	-20.0	100	2	0						
9	Primary drying	-17.0	100	0	20	✓					
10	Primary drying	-17.0	0	24	0						
11	Secondary drying	17.0	0	0	20	✓					
12	Secondary drying	17.0	0	18	0						
13	End of cycle										
14											
15											
16											
17											
18											

Figure S3. Recipe for cycle 3 used for the ramp temperature approach.

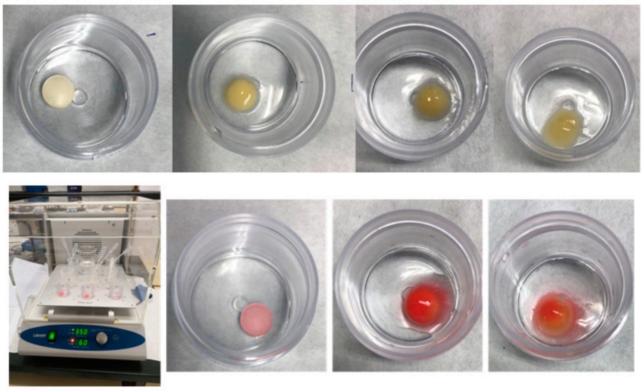

Figure S4. Tablets' aspect during swelling assay.

Table S1. Result from the palatability assay in healthy volunteers.

Formulation	Lip+L+A	Lip+L+C	Lip+L+A+C	Lip+L+M+A	Lip+L+M+C	Lip+L+M+A+C
Smell	3.2	3.3	2.5	2	3.7	3.7
Flavor	3.8	4.3	3.9	3.3	3.8	3.8
Texture	3.4	1.6	4.1	2.3	2.3	2.4
General feeling	3.2	3.3	3.9	2.7	2.8	3.3
Mean	3.4	3.1	3.6	2.6	3.2	3.3
S.D.	0.3	1.1	0.7	0.6	0.7	0.6

Table S2. Results from the in vivo assay performed to evaluate the sublingual suitability of tablets.

	Capsule-shaped tablets			Round tablets		
Participants	Adhesion time/ min	Adhesion Strength	Signs of irritation	Adhesion time/ min	Adhesion Strength	Signs of irritation
1	15	Non-adhesive/ Slightly adhesive when finished	non-irritating	52	Adhesive	non-irritating
2	26	Adhesive	non-irritating	50	Slightly adhesive/ Adhesive when finished	non-irritating
3	24	Slightly adhesive	non-irritating	50	Adhesive	non-irritating
4	30	Adhesive	non-irritating	40	Slightly adhesive	non-irritating
5	24	Slightly adhesive	non-irritating	35	Adhesive	non-irritating
6	34	Non-adhesive/ Slightly adhesive when finished	non-irritating	40	Adhesive	non-irritating
Mean±S.D.	25.50±6.44			45.40±7.47		
